# Small SNP panels for breed proportion estimation in Indian crossbred dairy cattle

**DOI:** 10.1111/jbg.12544

**Published:** 2021-03-09

**Authors:** Eva M. Strucken, Marimuthu Swaminathan, John P. Gibson

**Affiliations:** ^1^ Centre for Genetic Analysis and Applications School of Environmental and Rural Science University of New England Armidale NSW Australia; ^2^ BAIF Development Research Foundation Pune India

**Keywords:** admixture, ancestry, *Bos* *indicus*, *Bos* *taurus*, single nucleotide polymorphisms, smallholder

## Abstract

Reliably identifying breed proportions in crossbred cattle in smallholder farms is a crucial step to improve mating decisions and optimizing management in these systems. High‐density genotype information is able to estimate higher‐order breed proportions accurately, but, are too expensive for mass application in smallholder systems. We used high‐density genotype information (777 k SNPs) of 623 crossbred cattle from India that had Holstein‐Friesian (HFX) and/or Jersey and indigenous breeds in their ancestry to select a smaller number of SNPs for breed proportion estimation. The accuracy of estimates obtained from panels with 100–500 SNP was compared to estimates based on all SNPs. Panels were selected for highest absolute allele frequency difference between exotic dairy versus indigenous *Bos indicus*, or between HFX versus Jersey breeds. A step‐wise pruning approach was developed showing that and increased physical distances between markers of 8.5 Mb improved breed proportion estimation compared to a standard 1 Mb distance. A panel of 500 SNPs optimized to estimate HFX versus Jersey versus indicine ancestry was able to estimate indicine breed proportions with *r* ^2^ = .991, HFX proportions with *r* ^2^ = .979 and Jersey proportions with *r* ^2^ = .949. The number of markers was a deciding factor in estimation accuracy, together with the distribution of markers across the genome.

## 1 INTRODUCTION

In 1970, a dairy development program called Operation Flood saw large improvements and expansions of India's milk‐sheds to cover the countries large demand for milk and dairy products. Part of the program was to import high‐producing dairy cattle to cross with disease and heat resistant indigenous *Bos indicus* cattle (Singh, 1999). Most common exotic breeds were Holstein‐Friesian (HFX), Jersey and Brown Swiss which were mostly live animals but more often semen for artificial insemination were imported. The exotic genetics were sourced from many countries, and guidelines to maintain a level of exotic ancestry of approximately 50% were issued by the National Commission for Agriculture (Chacko, 1994). Crossbred cattle in India were recently shown to be an average of 70.3% (*SD* 9.6%) exotic ancestry (Strucken et al., 2018), thus showing a much larger exotic ancestry than initially favoured.

Since the 1950s, India has increased its milk production tenfold and is now the world's leading milk producer (Department of Animal Husbandry and Dairying, Cattle and Dairy Development Division). Crossbred cows contribute 54% of the total cattle milk production in India (Department of Animal Husbandry and Dairying (2019)). Most of the crossbred cows are kept on smallholder farms and accurate records of pedigrees and thus information about ancestry proportions are sparse. To further improve productivity of cattle in India, the BAIF Development Research Foundation has set out to evaluate breed compositions of Indian crossbred animals. Determining the breed composition of crossbred cattle and making this analysis affordable for smallholder farmers lays the foundation for an improved crossbreeding program including informed mating decisions.

Breed proportions of crossbred cattle can be accurately determined using high‐density genetic marker genotypes such as single nucleotide polymorphisms (SNPs). However, despite decreasing genotyping costs, even low‐density commercially available SNP arrays are still too expensive to be applied extensively in smallholder systems in developing countries, where cost is the major barrier for farmers to take part in breed improvement programs. Therefore, customized small SNP panels need to be developed to be used in India to be able to include the large majority of dairy producers. As few as 200 SNPs were found to be sufficient to determine the ancestry proportion of the two major cattle groups *B*. *indicus* and *Bos taurus* in African crossbred cattle (Strucken et al., 2017; Wilkinson et al., 2011). More markers, however, are required if ancestral breed proportions within *B. taurus* breeds should be distinguished. Gebrehiwot et al. (2021) showed that more markers are required to differentiate African from European taurine breeds compared to the differentiation of *B. indicus* and *B. taurus* in a ratio of 70%:30%.

Here, we refine the approaches to select SNPs for breed proportion estimation as outlined in Strucken et al. (2017) and Gebrehiwot (2020) by testing the influence of a step‐wise pruning of markers and a combination of best markers to estimate indicine breed proportions as well as individual HFX and Jersey proportions in 623 HFX and Jersey crossbred cattle from India.

## 2 MATERIAL AND METHODS

### 2.1 Data

**FIGURE 1:**
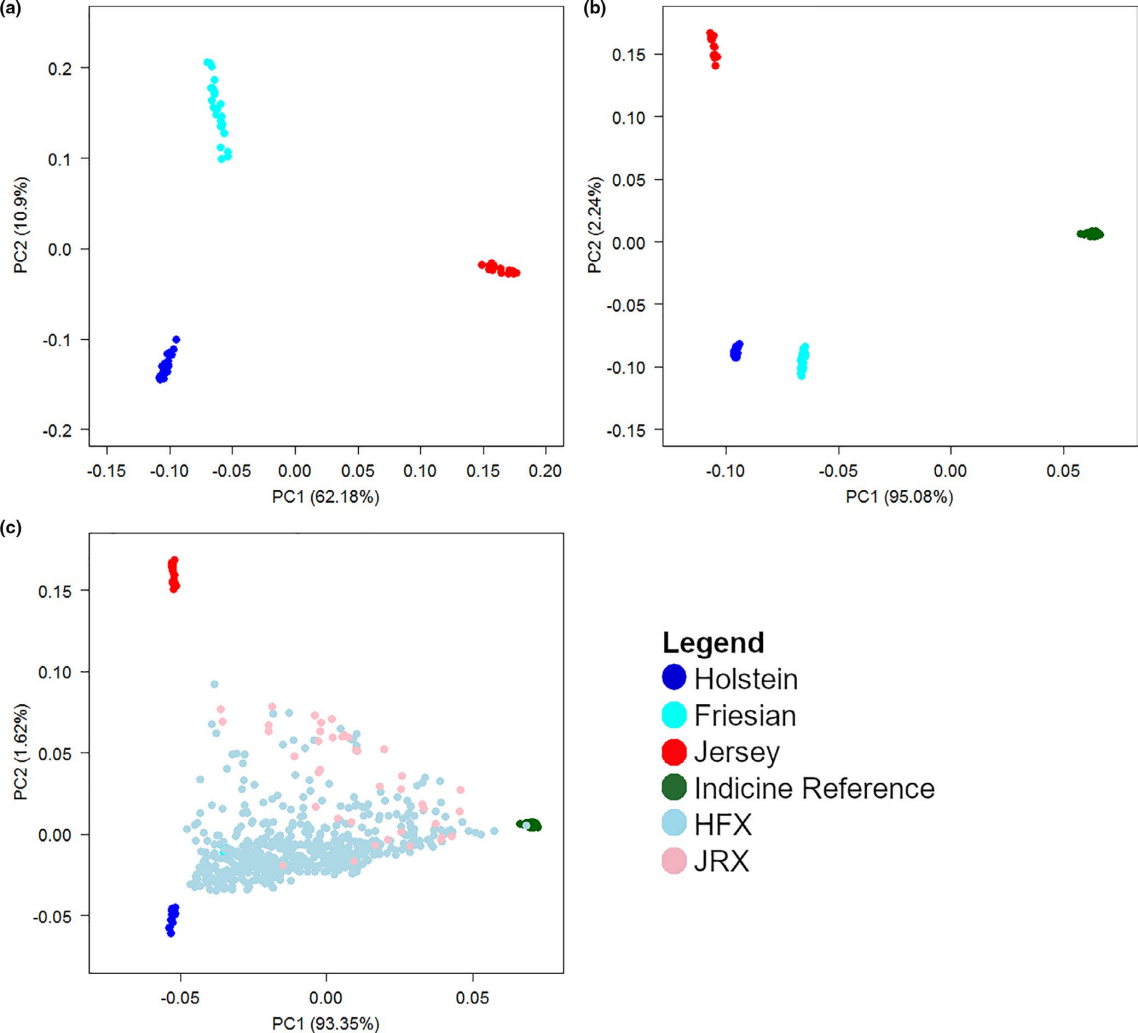
Principal components of (a) filtered exotic reference populations from HapMap and SRUC, (b) all reference populations, and (c) filtered reference populations and Indian dairy crossbreds

A total of 583 crossbred cattle classified in the field as HFX crosses and 40 classified as Jersey (JRX) crosses were provided by BAIF. They were sampled from smallholder dairy farmers in Maharashtra and Haryana. The large majority of crossbred cattle resulted from many generations of crossing between exotic and indigenous cattle and among crossbred with no pedigree recording, so that actual breed composition of the animals was not known at the time of sampling. Additionally, 525 Indian indigenous reference animals (IND) from 12 recognized breeds were available (Strucken et al., 2019), from which 101 animals were selected as an indicine reference sample. The indicine reference animals were selected to represent the least related individuals within breed, with highest average kinship with unselected individuals (Aliloo et al., 2018). The number of animals included was proportional to the total number of animals available for each breed. Exotic reference breeds of pure Holstein (H) and Jersey (J) animals were sourced from the bovine HapMap consortium (http://bovinegenome.org), and Friesian (F) animals from the Scottish Rural University College (SRUC) and reduced to 24 animals each that showed largest separation in a principal components plot (Figure 1).

All animals were genotyped with the 777 k SNP BovineHD Beadchip (Illumina Inc.). The data were quality controlled according to following parameters: genotype calls GC > 0.15, and call rates per marker and per animal >0.9. Animals were further checked for pair‐wise identity‐by‐state with a threshold of 0.98 to exclude duplicate samples. Only markers on autosomes were retained. The HapMap and SRUC data were obtained already quality controlled. After quality control and merging of data sets, 725,159 markers were included in analyses.

### 2.2 Principal components analysis

Principal components were calculated based on the genomic relationship matrix which was constructed according to Van Raden (2008). Missing genotypes were replaced by average genotypes across all animals.

$$\mathrm{GRM}={\mathrm{ZZ}}^{\prime }/2\times \sum p_{l}\times \left(1‐p_{l}\right),$$

where **Z** is the centred genotype matrix and p is the allele frequency at locus *l*. Matrix **Z** was constructed by subtracting from the genotype matrix **M** the **P** matrix, which equaled 2×(p −0.5). The centring of **Z** was achieved by subtracting −1 from **M**.

### 2.3 Observed and expected breed composition

Breed proportions of the crossbred animals were estimated with the ADMIXTURE 1.23 program (Alexander et al., 2009). The analysis was supervised with Jersey, Holstein, Friesian and the pooled indicine sample as assumed ancestral populations. We used all 725 k markers for breed proportion estimation to establish our observed breed proportions and to test the accuracy of the estimates from small SNP panels. The accuracy of breed proportion estimation of the small panels was calculated as the squared correlation between breed proportions estimated with all 725 k markers and the breed proportions estimated with the small panels. The linear bias of breed proportion estimates was calculated as the average deviation of estimates obtained with the marker subsets from the observed (725 k) breed proportions. Holstein‐Friesian (HF) proportion was defined as the sum of breed proportions for Holsteins or Friesians.

### 2.4 Selection of markers

Allele frequencies were calculated per exotic dairy breed (J, H, F), across all 72 exotic animals (EXO), across all Holstein and Friesian reference animals (HF), as well as for the indicine reference sample of 101 animals (IND). Absolute allele frequency differences were calculated between EXO versus IND, HF versus J and H versus F. Markers with largest absolute allele frequency differences were selected to create small SNP panels.

To remove markers that are likely to be in high linkage disequilibrium (LD) in the target populations, the data were pruned based on the physical distance between markers in the ancestral populations. The physical distance in the ancestral populations rather than a direct LD measure in the target population was chosen to have a population independent pruning, ensuring that the selected marker panels can be applied in different target populations of a similar ancestral background. We started the pruning at a distance of 1 Mb, as this corresponds approximately to 1 cM in mammals which assumes an average of 0.01 chromosomal crossovers per generation. We further increased this physical distance in 0.5 Mb steps to a maximum of 10 Mb.

The larger the pruning distance between markers the fewer markers could be selected from the total data set of 725 k markers. Therefore, we created a step‐wise pruning approach starting at the distance which yielded the highest breed proportion accuracy with 100 markers and decreasing the distance thereafter. For the EXO versus IND allele frequency differences, the first 100 markers were pruned for 8.5 Mb, the next 100 for 7.5, then 6.5, 4.5 and 3.5 Mb. For the HF versus J allele frequency differences, the first 100 markers were pruned for 5 Mb, the next 200 markers for 4 Mb and another 200 markers for 3 Mb. The accuracies for individual Holstein or Friesian breed proportions were under 0.784 with 100 markers, which is why we decided not to pursue individual breed proportion estimation for these breeds.

Previous studies have shown that 200–400 markers are sufficient to obtain accurate estimates of dairy breed proportions (Gebrehiwot et al., 2021; Strucken et al., 2017), and that only a negligible improvement in estimation accuracy was achieved with more than 500 markers. Therefore, we tested small panels of 100, 200, 300, 400 and 500 SNPs.

The distinction between *B. taurus* (EXO) and *B. indicus* (IND) can be achieved with relatively fewer markers compared to the distinction between *B. taurus* breeds (Gebrehiwot et al., 2021). To develop a panel that could distinguish between indicine and exotic as well as between Holstein‐Friesian and Jersey proportions, we created panels with different ratios of markers selected from both optimized panels for EXO versus IND and HF versus J. We compared ratios of EXO versus IND:HF versus J from 50:50 to 10:90 in 5‐step increments. Five panel sizes were tested, varying from 100 to 500 SNPs per panel. Figure S1 shows a flow chart of the selection process.

The data specifically collected for this study can be obtained upon reasonable request from BAIF. Contact information is provided at the end of the manuscript. The ranked list of SNPs identified to provide best breed proportion predictions as described in this manuscript can be found in Tables S1,S2. The SNPs are in rank order based on absolute allele frequency difference between the two target ancestor populations.

## 3 RESULTS AND DISCUSSION

### 3.1 Data structure

Principle component analyses were used to select Holstein, Friesian, and Jersey animals that clustered in distinct groups and would therefore have largest genetic differences between the three exotic reference breed populations. Figure 1a shows the PC plot for exotic dairy breeds sourced from HapMap after filtering. The largest genetic differences were found between the Jersey and the Holstein/Friesian group with PC1 explaining 62.18% of the genetic variance, while PC2 separated the Holsteins from the Friesians and explained 10.9% of the genetic variance (Figure 1a). When including the indicine reference group, the differentiation between the exotic taurine dairy breeds and the indicine reference group explained 95.08% of the genetic variance (Figure 1b). Accurate breed proportion estimation for Holstein and Friesian separately is most likely less accurate compared to total exotic dairy proportion, as the genetic differences are comparably smaller.

The crossbred animals spread between the Indian indigenous sample and the Holstein‐Friesian and Jersey clusters. Notably, most HFX aligned reasonably close to the indigenous‐HF axis. The remaining animals, including most animals classified as JRX, aligned between the HF‐indigenous and J‐indigenous axes, indicating that they had both HF and J ancestry (Figure 1c). The alignment of the crossbred animals with the exotic dairy breeds is not perfect and could potentially be improved in the future if genetic samples of originally imported Jersey, Holstein and Friesian breeds from the 1970 become available. Nonetheless, SNPs selected from the current reference samples are unbiased due to the reference being independent from the target population. The small sample size of the reference population could lead to an inaccurate identification of SNPs with highest allele frequency differences between ancestral breeds; however, the estimates of accuracy in the target population remain unbiased.

**FIGURE 2:**
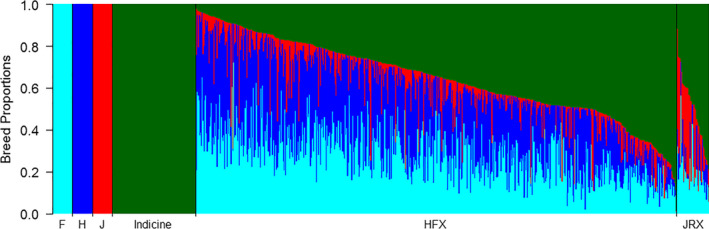
Breed proportions of Indian Holstein‐Friesian (HFX) and Jersey (JRX) crossbreds from a supervised analysis with four ancestral breeds and 725 k SNPs

The admixture of the HFX crossbreds ranged from 0.02% to 98.8% indicine breed proportion, and the JRX crossbreds ranged from 11.1% to 79.6% indicine breed proportion (Figure 2). The HFX crossbreds had average Holstein proportions of 29.9% (*SD* 14.2%), Friesian proportions of 28.2% (*SD* 12.3%) and Jersey proportions of 6.7% (*SD* 8.7%). Twenty‐six HFX (4.5%) had Jersey proportions >25%, of which one animal had 60% Jersey proportion. The JRX crossbreds had an average Jersey proportion of 24% (*SD* 17.3%), Holstein proportion of 7.6% (*SD* 6.6%) and Friesian proportion of 19.1% (*SD* 9.2%). 17 JRX (42.5%) had Holstein‐Friesian proportions >25%, with two animals having >50% HF proportions. The results illustrate the wide range of admixture of crossbreds, in terms of indicine versus taurine ancestry and in terms of mixed history of Holstein‐Friesian and Jersey ancestry in animals that visually appear to be HFX or JRX.

Crum et al. (2019) pointed out several sources for changing estimates in ADMIXTURE. We carried out internal checks on data and model‐related differences of estimates in ADMIXTURE and found that higher‐order estimates, such as total exotic or indicine proportions, are highly robust, irrespective of number of reference breeds, number or order of target animals. Further, Alexander and Lange (2011) report that breed proportion estimates became more accurate with an increase in *F*_ST_ from 0.01 to 0.05. Our internal checks confirm that populations need to be sufficiently genetically differentiated for ADMIXTURE to produce robust estimates, and we even found that *F*_ST_ values should ideally be >0.1 (results not shown). In the present study, the global *F*_ST_ value between Jersey and Holstein was 0.16, between Jersey and Friesian 0.137, and between Holstein and Friesian 0.074, which indicates that ADMIXTURE should be able to differentiate between Jersey and Holstein‐Friesians, but might have problems differentiating between the closer related populations of Holstein and Friesian. Crum et al. (2019) do not report *F*_ST_ values for the breeds where they observed changing ADMIXTURE estimates.

### 3.2 Distribution of SNPs

**FIGURE 3:**
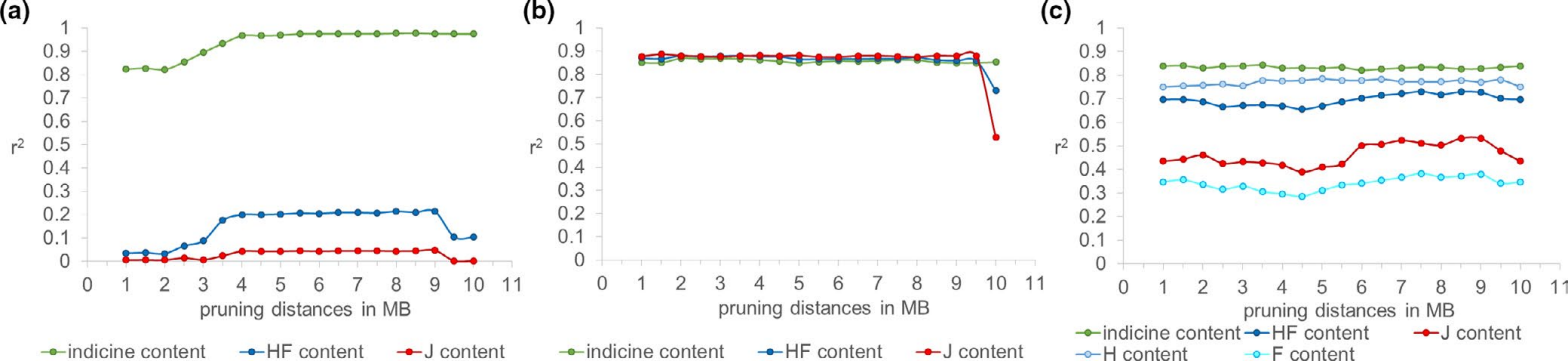
Breed proportion accuracy for 100 markers at different pruning distances in all crossbreds for (a) largest absolute allele frequency difference EXO versus IND, (b) HF versus J and (c) H versus F

When selecting markers for breed proportion estimation, it has been shown that higher accuracies are achieved the more evenly distributed the markers are across the entire genome (Gebrehiwot, 2020). The top 500 markers sorted by absolute allele frequency difference between exotic and indicine reference breeds showed substantial clustering of markers, even when applying a minimum 1 Mb pruning distance between markers (Figure S2). The highest estimation accuracy of 0.977 for indicine content with 100 markers was observed for a pruning distance of 8.5 Mb, although between 4 and 10 Mb, estimation accuracies were only minimally different for the top 100 markers (Figure 3a). Applying smaller distances resulted in a drop of accuracy with the lowest accuracy of 0.822 found for 2 Mb. A similar drop in accuracies was found for estimating individual Holstein‐Friesian or Jersey content, and the estimation accuracy of these breed contents also experienced a drop with pruning distances >9 Mb (Figure 3a).

Using the absolute allele frequency differences between HF and J increased the estimation accuracy of Holstein‐Friesian and Jersey content with 100 markers to a maximum of 0.88 (3.5 Mb pruning) and 0.886 (1.5 Mb pruning), respectively. Again, differences in estimation accuracies were minimal between pruning differences but a drop in accuracies was observed with distances >9.5 Mb (Figure 3b). The accuracy for Jersey content was highest (*r* ^2^ = .956) when only the JRX were considered and a 5 Mb pruning distance applied (not shown), which is why we chose this distance as a starting point for the step‐wise pruning approach explained later.

Trying to improve estimation accuracy for the individual Holstein and Friesian proportions, absolute allele frequency differences between H and F were used to select SNPs. Highest accuracies for Holstein proportions of 0.784 at 5 Mb pruning distance and for Friesian proportions of 0.383 at 7.5 Mb pruning distance were found (Figure 3c).

Large allele frequency difference between the reference populations gives the highest estimation accuracy for the ancestral breed proportions in the target population. When pruning a list of SNPs that have been ranked by allele frequency difference, those SNPs chosen later in the process have lower absolute allele frequency differences. Similarly, as the pruning distance increases, the absolute allele frequency difference of chosen SNPs will decrease.

**FIGURE 4:**
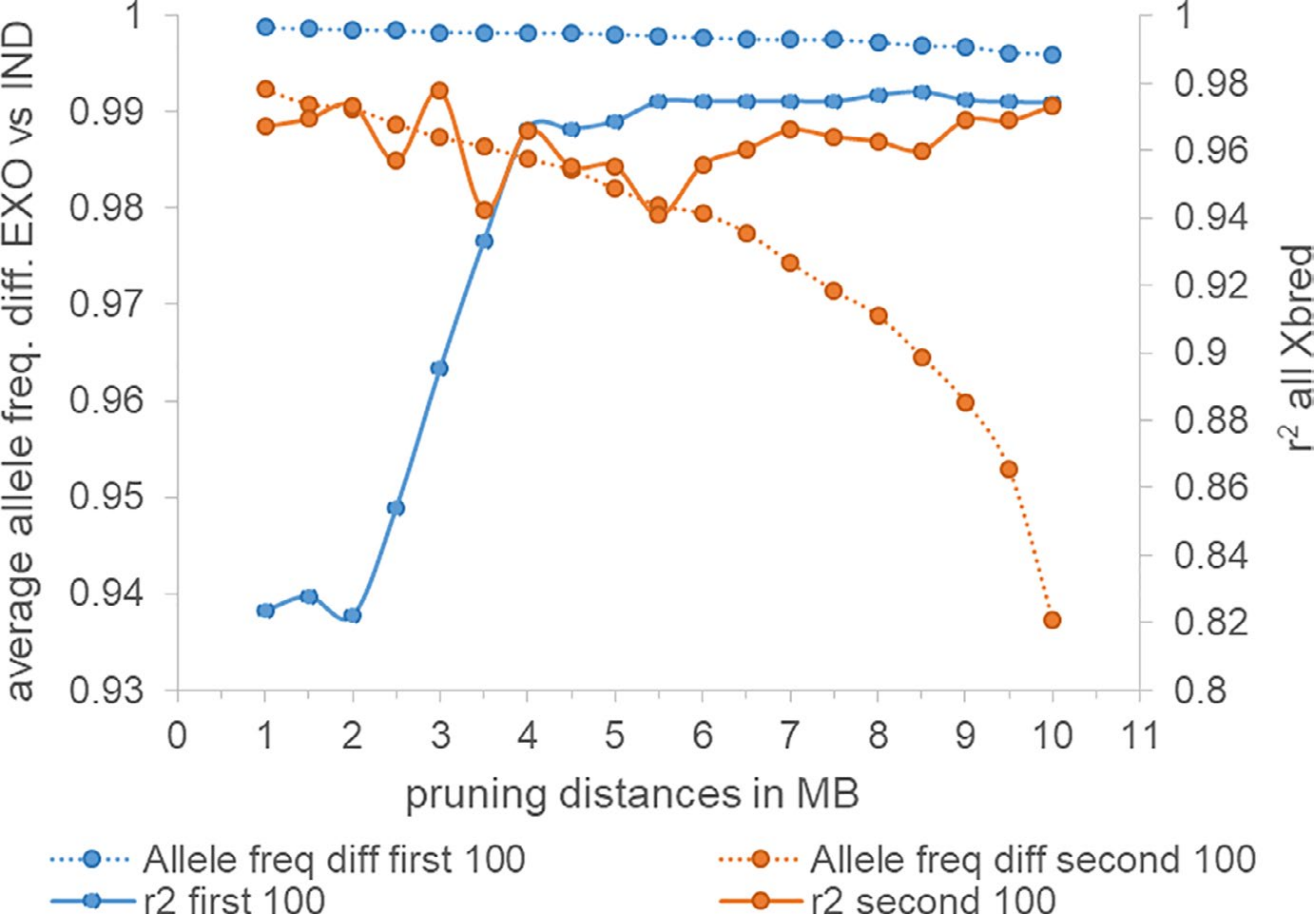
Average allele frequency difference and estimation accuracy of indicine breed proportions for the first (blue) and the second 100 markers (orange) selected at different pruning distances, for panels designed to estimate exotic versus indicine ancestry

Examining the allele frequency differences for the first 100 markers between EXO versus IND in relation to pruning distance and achieved estimation accuracy shows that allele frequency differences are >0.99 at all pruning distances; however, the estimation accuracy is lower for smaller pruning distances (Figure 4). This demonstrates that for the top 100 markers, achieving a distribution across the genome that avoids high LD between markers in the target population drives estimation accuracy.

Considering the next 100 markers (markers 101–200), confirms the expectation that larger pruning distances eventually result in marker panels with lower absolute allele frequency differences, reaching 0.937 at 10 Mb pruning distance. Nevertheless, estimation accuracies do not show an equal drop. In fact, estimation accuracies are highest either with low or high pruning distances (Figure 4). Therefore, we conclude that absolute allele frequency differences between ancestral breeds have to be much lower than the observed 0.937 to impact estimation accuracies in the target population.

### 3.3 Impact of panel size

For the estimation of exotic versus indicine breed proportions, a pruning distance of 8.5 Mb for the first 100 markers achieved the highest estimation accuracy. Starting with a pruning distance of 8.5 Mb for the first 100 markers, we lowered pruning distance in a step‐wise manner, selecting the best 100 markers at every step until 500 markers were selected. Final pruning distances were 7.5, 6.5, 4.5 and 3.5 Mb. These distances were chosen to keep the pruning distance as high as possible but at the same time being able to select an additional 100 markers at each step (Table S1). Other combinations of pruning distances were tested, but the described combination achieved highest accuracies.

Similarly, a step‐wise pruning method was applied for the estimation of HF versus J; however, the starting distance was 5 Mb. We chose 5 Mb as Jersey breed proportions were best estimated in JRX with this distance and because there was not much difference in accuracies with other distances. The first 100 markers were pruned with 5 Mb distance, the following 200 markers with 4 Mb distance and the last 200 markers with 3 Mb distance (Table S2).

The distribution of the 500 markers across the genome selected for EXO versus IND and HF versus J are shown in Figure S3.

**FIGURE 5:**
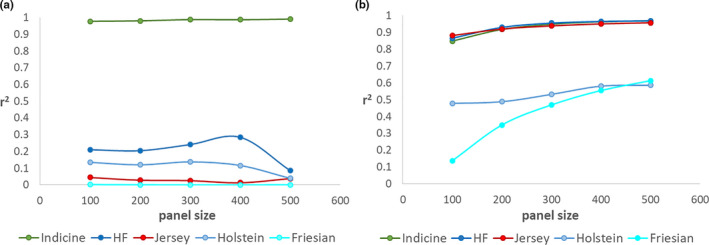
Estimation accuracies for different ancestral breeds in all crossbreds based on (a) step‐wise pruning for EXO versus IND, (b) step‐wise pruning for HF versus J

Indicine breed proportions were estimated with an accuracy of 0.977 with 100 (linear bias 0.014, *SD* 0.028) and 0.991 (linear bias 0.009, *SD* 0.018) with 500 markers in all crossbreds based on step‐wise pruning and absolute allele frequency differences based on EXO versus IND. All other breed proportions were estimated with an accuracy <0.3 (Figure 5a) and an absolute linear bias >0.12. Estimation accuracy was improved for Holstein‐Friesian and Jersey proportions when selection was based on absolute allele frequency difference between HF versus J. With 100 markers, Holstein‐Friesian and Jersey proportions were estimated with an accuracy of 0.865 (bias 0.012, *SD* 0.075) and 0.881 (bias −0.014, *SD* 0.036), respectively, and with 500 markers with an accuracy of 0.968 (bias 0.018, *SD* 0.037) and 0.957 (bias −0.009, *SD* 0.022), respectively (Figure 5b). Estimation accuracy for indicine breed proportion was lower than when SNPs were chosen based on EXO versus IND, being 0.848 and 0.968 for 100 and 500 markers, respectively.

Combining the top 500 markers for EXO versus IND and HF versus J into a 1,000 SNP panel resulted in estimation accuracies for indicine breed proportions of 0.993, for Holstein‐Friesian breed proportions of 0.979 and for Jersey breed proportions of 0.945.

Estimation accuracies were already low for optimized panels to distinguish Holstein from Friesian proportions. Merging the top 500 markers from each of the three breed comparisons and using 1,500 markers, accuracies only achieved an *r* ^2^ of .905 and .824 for Holstein and Friesian proportions, respectively. Another improvement to distinguish these closely related breeds could potentially be made with a different Friesian sample, as the available sample here had 13.5% of missing genotypes across all 24 samples while the other breed samples had <0.22% missing genotypes.

We combined the two optimized panels for EXO versus IND and HF versus J in different ratios to maintain a high estimation accuracy for all three ancestral breeds but lower the number of SNPs required. This combination of optimized panels was favoured over alternative approaches, such as using the fixation index *F*_ST_, where markers could be selected to distinguish more than two ancestors at once, for example distinguish between IND, HF and J. This is because such measures will be dominated by the vastly greater genetic distance between indigenous (*B. indicus*) versus exotic dairy (*B. taurus*), such that ranking of SNPs based on three‐way measures of diversity, such as *F*_ST_, would not be expected to identify SNPs that effectively discriminate HF from J ancestry.

**FIGURE 6:**
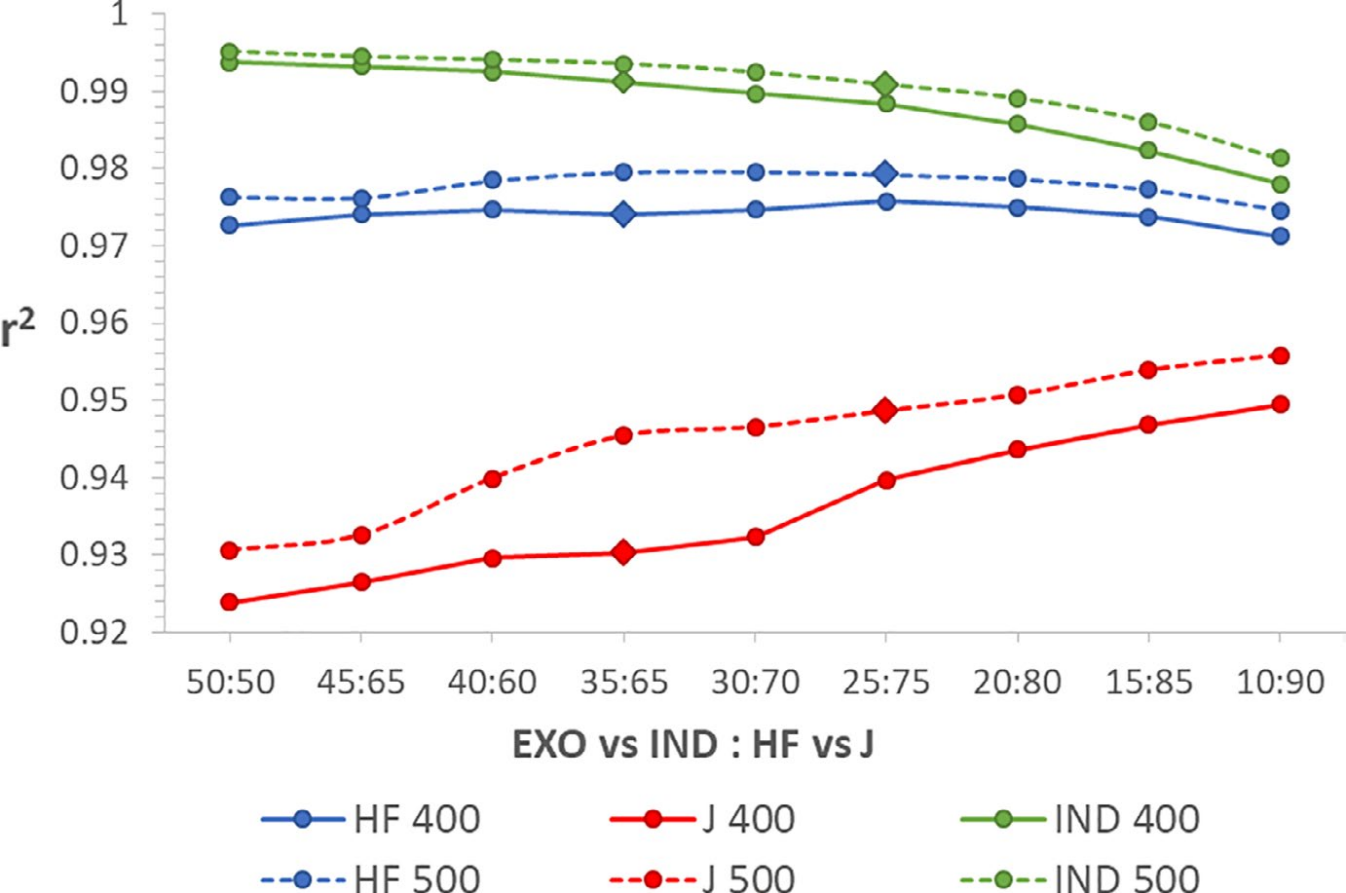
Estimation accuracies for panels selected with different ratios from the two optimum panels for 400 and 500 SNPs

It has been previously shown that the large genetic difference between *B. taurus* and *B. indicus* requires fewer markers to estimate these breed proportions compared to estimating breed proportions from less distant breeds (Gebrehiwot et al., 2021). Therefore, we only tested a 50:50 ratio or ratios with lower proportions of markers from the EXO versus IND SNP‐set. Maintaining an estimation accuracy >0.99 for indicine breed proportion and increasing the accuracy for Holstein‐Friesian and Jersey estimation resulted in an optimum combined panel of 500 markers at a ratio of 25% EXO versus IND and 75% HF versus J. With this panel, indicine breed proportions were estimated with an accuracy of 0.991 (linear bias 0.002, *SD* 0.018), which is only 0.002 units lower than the estimation accuracy with 1,000 markers; the Holstein‐Friesian proportion was estimated with an accuracy of 0.979 (0.0002 lower than with 1,000 markers), and a linear bias of 0.008 (*SD* 0.03); and the Jersey proportion was estimated with an accuracy of 0.949 (0.004 higher than with 1,000 markers; Figure 6), and a linear bias of −0.01 (*SD* 0.024). Lowering the number of markers to 400 and keeping the estimation accuracy for indicine breed proportion >0.99 is possible with a ratio of 35:65; however, the estimation accuracies for Holstein‐Friesian and Jersey proportion drop by 0.005 and 0.018, respectively, compared to the 500 marker panel (Figure 6), and the linear bias of the estimates remained almost unchanged. The linear bias of the combined 500 (25:75) and 400 (35:65) panels is shown as regression plots in Figure S4. No combination of markers from both optimum panels allowed for lowering the number of markers further without decreasing estimation accuracy for indicine breed proportion under 0.99.

Both optimum panels are provided in Tables S1,S2 and can be combined in the suggested ratios to create custom breed proportion panels to determine indicine, Holstein‐Friesian and Jersey proportions in crossbred cattle know to stem from these ancestral breeds. The supplemental tables are ranked, and SNPs should be chosen from the top down.

The estimates of breed proportions in the cows classified as HFX had slightly lower accuracy than those classified as JRX (Table 1). Excluding HFX cows that had a high Jersey content and JRX cows that had a high Holstein‐Friesian content resulted in a drop in *r* ^2^ but had very little effect of the *SE* of estimates of breed proportions. There was a relatively small increase in accuracy moving from the optimum set of 400 SNP to the optimum set of 500 SNP.

**TABLE 1:** Breed proportion estimation accuracy measured as the *SE* of the estimate and as *r* ^2^ for two small SNP panels in Holstein‐Friesian crossbreds (HFX) and Jersey crossbreds (JRX) depending on purity of the cross

SNP panel	Breed proportion	HFX			JRX		
		All HFX	J < 50%	J < 30%	All JRX	HF < 50%	HF < 30%
400 (ratio 35:65)	Indicine	0.991 (0.018)	0.991 (0.018)	0.991 (0.018)	0.992 (0.015)	0.993 (0.015)	0.993 (0.014)
	HF	0.971 (0.033)	0.971 (0.033)	0.970 (0.032)	0.944 (0.031)	0.910 (0.031)	0.709 (0.028)
	Jersey	0.906 (0.027)	0.901 (0.027)	0.822 (0.025)	0.981 (0.024)	0.979 (0.024)	0.987 (0.019)
500 (ratio 25:75)	Indicine	0.990 (0.018)	0.990 (0.018)	0.990 (0.018)	0.994 (0.013)	0.995 (0.013)	0.995 (0.012)
	HF	0.977 (0.029)	0.977 (0.029)	0.977 (0.028)	0.953 (0.029)	0.923 (0.029)	0.762 (0.026)
	Jersey	0.930 (0.023)	0.926 (0.023)	0.867 (0.021)	0.988 (0.019)	0.986 (0.020)	0.990 (0.016)

Abbreviation: HF, Holstein‐Friesian; J, Jersey.

In some regions of India, though not in the areas from which our crossbred samples were taken, Brown Swiss cattle were imported for crossbreeding purposes (Chacko, 1994). Melka and Schenkel (2012) observed that fixation index *F*_ST_ between North American Holstein and Jersey was lower than that between North American Brown Swiss and either Holstein or Jersey. Signer‐Hasler et al. (2017) looked at the genetic differentiation between Swiss dairy cattle and also found the largest differentiation between Brown Swiss and Holstein; however, they did not include Jersey cattle. This larger genetic differentiation indicates that SNPs could likely be selected to successfully identify an ancestral Brown Swiss component in Indian crossbred cattle that could achieve estimation accuracies similar to those found for Holstein‐Friesian and Jersey proportions in the present study.

## 4 CONCLUSION

The informativeness of a small SNP panel for distinguishing breed ancestries in cattle largely depends on the number of markers, the distribution across the genome to avoid LD between markers in the target population, and the genetic differentiation between the ancestral breeds. The number of markers is sought to be as low as possible without losing estimation accuracy in order to increase the routine uptake of genomic tools to estimate breed proportions where financial resources are limited. The distribution across the genome and the distance between markers was shown to play a major role in the performance of the small SNP panels, and a pruning distance of 1 Mb was shown to be too short, resulting in a considerable clustering of markers which reduced estimation accuracy. The very large genetic differentiation between *B. taurus* and *B. indicus* breeds allows accurate estimation of *B. indicus* versus *B. taurus* ancestry with relatively few markers. The lesser genetic differentiation between *B. taurus* breeds, in our study Holstein‐Friesian versus Jersey, requires more informative marker to achieve a useful estimation accuracy. We designed an optimized panel of 500 SNPs to differentiate indigenous from Holstein‐Friesian from Jersey ancestry in the majority of populations in India, where only these two exotic breeds have been used. An assay that includes estimation of Brown Swiss ancestry for use in other areas of India could likely be developed.

## CONFLICT OF INTEREST

The authors declare that they have no competing interests.

## AUTHOR CONTRIBUTIONS

Analyses were designed by EMS, JPG and MS, performed by EMS and interpreted by EMS, JPG and MS. The manuscript was written by EMS and JPG, with inputs from all other authors.

## ETHICAL APPROVAL

The samples collected for this study involved prior informed consent of the owners of the cattle, and blood samples were collected by veterinarians using the minimum‐harm protocols that apply to blood sampling for routine disease surveillance and diagnostics.

## Supporting information

Fig S1

Fig S2

Fig S3

Fig S4

Table S1

Table S2

## Data Availability

The data generated specifically for this study are available from M. Swaminathan mswami@baif.org.in on reasonable request. Reference data are available from public and private databases as detailed in the paper.
