# Supplementary figures and images for: Small SNP panels for breed proportion estimation in Indian crossbred dairy cattle
Source: J Anim Breed Genet. 2021 Mar 9;138(6):698–707. doi: 10.1111/jbg.12544 (PMC8519156; doi:10.1111/jbg.12544)

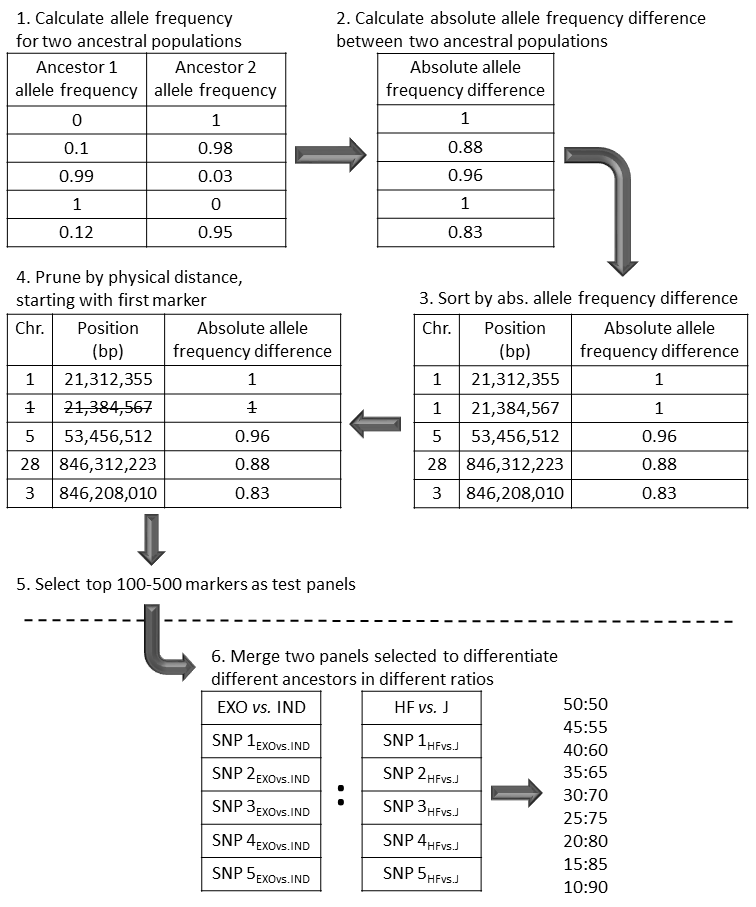

Supplement: Supplementary file 1 — Fig S1 [file JBG-138-698-s005.tif]

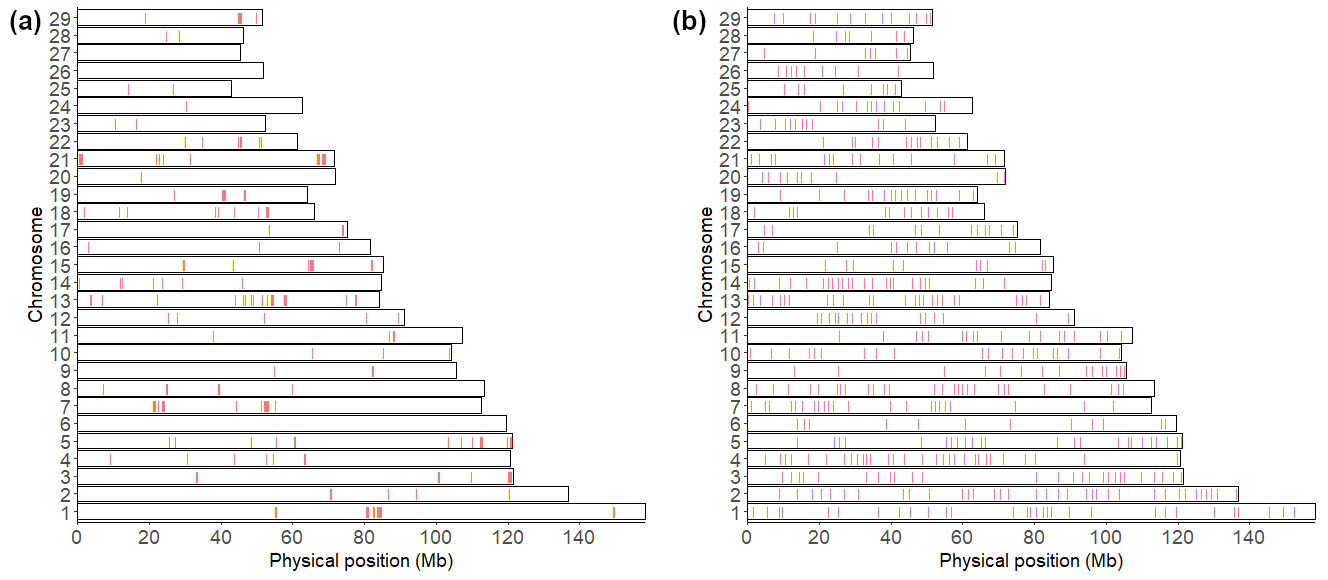

Supplement: Supplementary file 2 — Fig S2 [file JBG-138-698-s002.tiff]

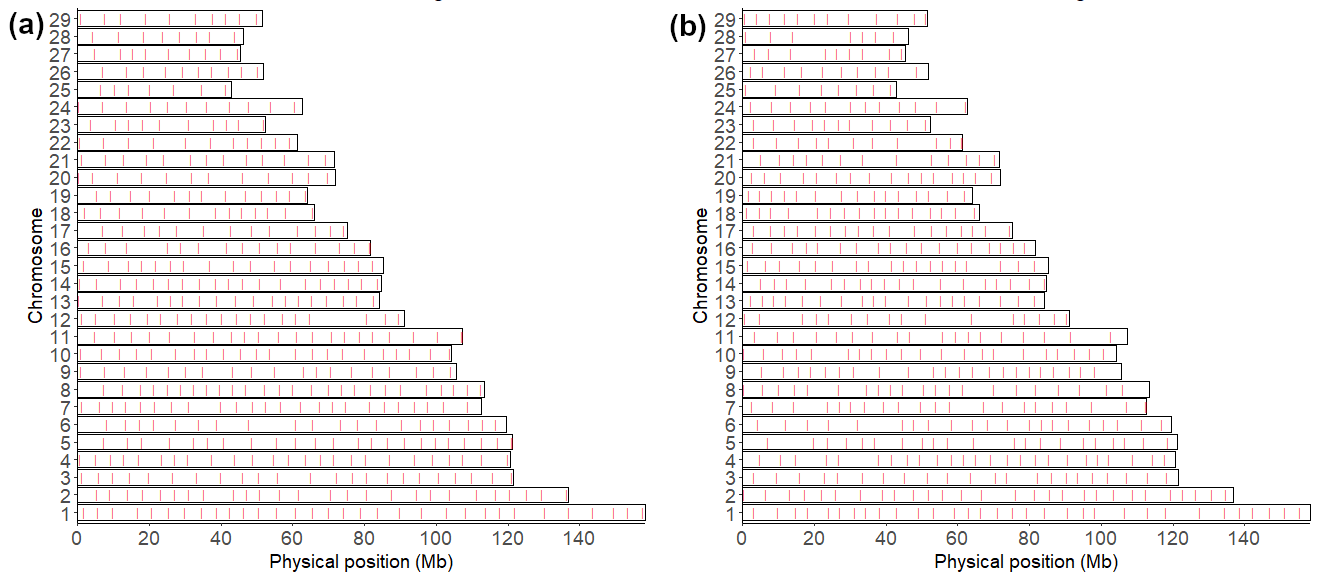

Supplement: Supplementary file 3 — Fig S3 [file JBG-138-698-s004.tiff]

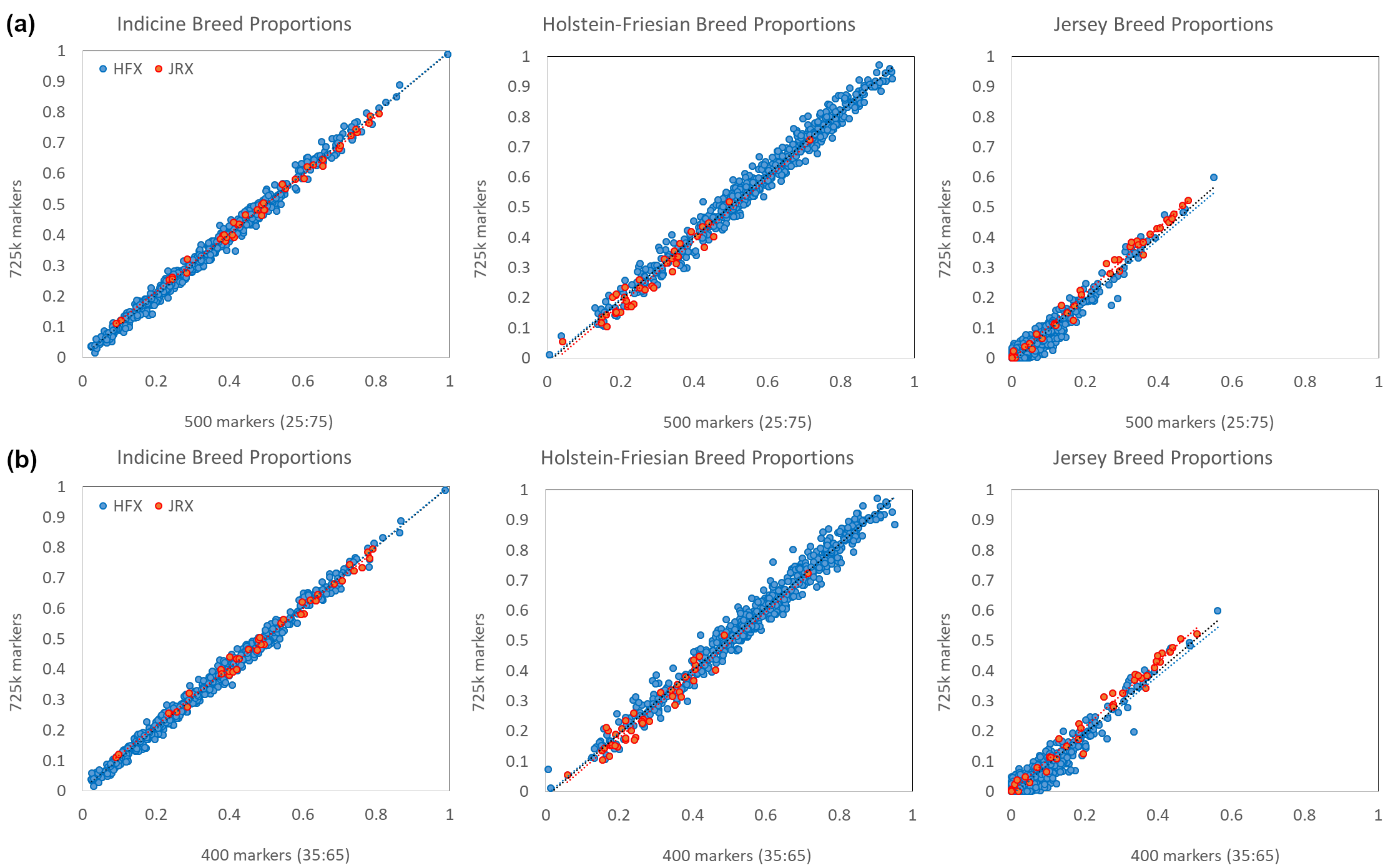

Supplement: Supplementary file 4 — Fig S4 [file JBG-138-698-s003.tiff]
